# Protocol for the Support Application for Food PAntrieS trial: design, implementation, and evaluation plan for a digital application to promote healthy food access and support food pantry operations

**DOI:** 10.3389/fpubh.2024.1340707

**Published:** 2024-05-24

**Authors:** Daniel J. Barnett, Samantha M. Sundermeir, Melissa M. Reznar, Alexis Lightner, Lisa Poirier, Andrew J. Rosenblum, Ayoyemi Toheeb Oladimeji, Takeru Igusa, Roni Neff, Cara F. Ruggiero, Emma C. Lewis, Leah Jager, Leena Moses, Veronica Velez-Burgess, Brooke Gagnon, Noor Attar, Joel Gittelsohn

**Affiliations:** ^1^Department of Environmental Health and Engineering, Johns Hopkins University Bloomberg School of Public Health, Baltimore, MD, United States; ^2^Department of International Health, Johns Hopkins University Bloomberg School of Public Health, Baltimore, MD, United States; ^3^School of Health Sciences, Oakland University, Rochester, MI, United States; ^4^Public Health Studies, Johns Hopkins University Krieger School of Arts and Sciences, Baltimore, MD, United States; ^5^Department of Civil and Systems Engineering, Johns Hopkins University Whiting School of Engineering, Baltimore, MD, United States; ^6^Division of General Academic Pediatrics, Department of Pediatrics, Mass General for Children, Boston, MA, United States; ^7^Division of Public Health Sciences, Fred Hutchinson Cancer Center, Seattle, WA, United States; ^8^Behavioral and Community Health, School of Public Health, University of Maryland, College Park, MD, United States

**Keywords:** food pantries, food pantry clients, charitable food system, app, digital health, food insecurity, urban, emergency operations center

## Abstract

**Introduction:**

Food-insecure households commonly rely on food pantries to supplement their nutritional needs, a challenge that was underscored during the COVID-19 pandemic. Food pantries, and the food banks that supply them, face common challenges in meeting variable client volume and dietary needs under normal and emergency (e.g., pandemic, natural disaster) conditions. A scalable digital strategy that has the capacity to streamline the emergency food distribution system, while promoting healthy food options, managing volunteer recruitment and training, and connecting to emergency management systems in times of need, is urgently required. To address this gap, we are developing a working mobile application (app) called the Support Application for Food PAntrieS (SAFPAS) and will evaluate its feasibility and impact on food pantry staff preparedness, stocking, and client uptake of healthful foods and beverages in two urban United States settings.

**Methods:**

This paper describes the protocol for a randomized controlled trial of the SAFPAS mobile application. We will conduct formative research in Baltimore, Maryland and Detroit, Michigan to develop and refine the SAFPAS app and increase scalability potential to other urban settings. Then we will test the app in 20 food pantries in Baltimore randomized to intervention or comparison. The impact of the app will be evaluated at several levels of the emergency food system, including food pantry clients (*n* = 360), food pantry staff and volunteers (*n* = 100), food pantry stock, and city agencies such as the local food bank and Office of Emergency Management. The primary outcome of the SAFPAS trial is to improve the healthfulness of the foods received by food pantry clients, measured using the Food Assessment Scoring Tool (FAST). Post-trial, we will conduct additional formative research in Detroit to prepare the app for scale-up.

**Discussion:**

We anticipate that SAFPAS will improve alignment in the supply and demand for healthy foods among food pantry clients, food pantries, and city agencies which supply food in Baltimore. Real-time, bidirectional communication between entities across the system allows for increased situational awareness at all levels during normal and emergency operations. By conducting formative research in Detroit, we hope to increase the scalability of the SAFPAS app to additional settings nationwide.

**Clinical trial registration:**

NCT87654321. https://classic.clinicaltrials.gov/ct2/show/NCT05880004.

## 1 Introduction

In 2021, about 13.5 million households (10.2%) in the United States (US) reported experiencing food insecurity at some point during the year (1). Food-insecure households may rely on food pantries to supplement their nutritional needs. While US food insecurity prevalence remained stable during the pandemic, use of food pantries increased from 4.4% in 2019 to 6.7% in 2020 (2, 3). Among food-insecure households, 36.5% reported using a food pantry, and among households with a very low food security, 45.5% reported using a food pantry in 2020 (3). An increased need during the pandemic underscored the challenges commonly faced by food pantries, and the food banks that supply them, in meeting variable client volume and dietary need (4). For example, food pantries tend to have limited staff capacity and resources to provide a safe forum for client choice and to assist with making healthy selections (5, 6). Additionally, food pantries are largely limited in what they can offer by what they receive from the central food banks, and thus tend to stock fewer healthy options despite their desire to emphasize healthy foods (7). As a result, previous studies have indicated that food pantry clients have lower diet quality (8).

To this end, capacity building and communication are necessary at multiple levels of the emergency food system to better align the supply and demand for healthier food options at food pantries. There is currently no system in place to support bidirectional information flow from clients up through city-level agencies. Thus, it is difficult to communicate client preferences and food pantry needs to food banks and city agencies who can help direct resources to food pantries.

This critical need during normal operations becomes even more pronounced during emergency situations (e.g., pandemic, natural disaster) to ensure access to consistent, nutritionally adequate food for pantry clients, and to provide real-time, data-driven situational awareness (5) of population health vulnerabilities. For example, personal communications between the research team and Baltimore City food pantries and city agencies revealed that city agencies and clients had no way of knowing which food pantries were open during the pandemic, needed supplies like PPE, or could accept new clients.

Digital strategies offer a promising solution given the ubiquity of smart phone use and evidence from multiple fields of research that digitizing can streamline operations and communications (9–12). The emergency food system has already started to move in this direction: food banks often have online reporting portals for food pantries, and other apps exist to address client tracking and inventory management (12, 13). However, many food pantry operations (e.g., volunteer management, training, and communication, client choice, and communications with city agencies) are outside the scope and capabilities of existing applications, and there is no “one-stop-shop” that houses all necessary functions (12). Importantly, there is interest among food pantry directors nationwide for a digital solution to enhance pantry management and operations (14).

Thus, a scalable digital strategy that promotes healthy foods at multiple levels of the emergency food system, streamlines food distribution efforts at each level, provides a mechanism for volunteer recruitment and training, enhances access to healthy foods, and connects with emergency management systems in times of need, is urgently required. The timeliness of such a digital strategy is further underscored by the lessons learned from COVID-19 and the looming expectancy of future disasters and emergencies. To fill this gap, we are developing a Support Application for Food PAntrieS (SAFPAS or “safepass”) working mobile application (app), and evaluating its feasibility and impact on food pantry staff preparedness, stocking, and client uptake of healthful foods and beverages. In this paper, we describe the protocol for this randomized controlled trial of the SAFPAS mobile application. The aims of the SAFPAS trial are to:

1. Develop a technically functional digital tool to improve food pantry services in low-income food settings;
2. Implement a randomized controlled trial of the SAFPAS app in Baltimore, Maryland and assess its feasibility;
3. Evaluate the impact of the SAFPAS app on the healthiness of foods received, food security, and food-related psychosocial factors by food pantry clients in a sample population of low-income urban clients in Baltimore, Maryland; and
4. Evaluate the impact of SAFPAS on food pantry stocking, staff capacity, and emergency preparedness and response in Baltimore, Maryland.

Formative research will be conducted in Detroit, Michigan pre- and post-trial to refine the app and increase the potential scalability to other urban settings outside of Baltimore, Maryland in a future, NIH R01 trial. Detroit was selected given existing partnerships with researchers, food banks, and food pantries in that area, and the sociodemographic similarities with Baltimore, and will be included in a future trial. Funding limitations did not allow piloting of the app in multiple localities.

## 2 Methods

### 2.1 Overview

**Figure 1:**
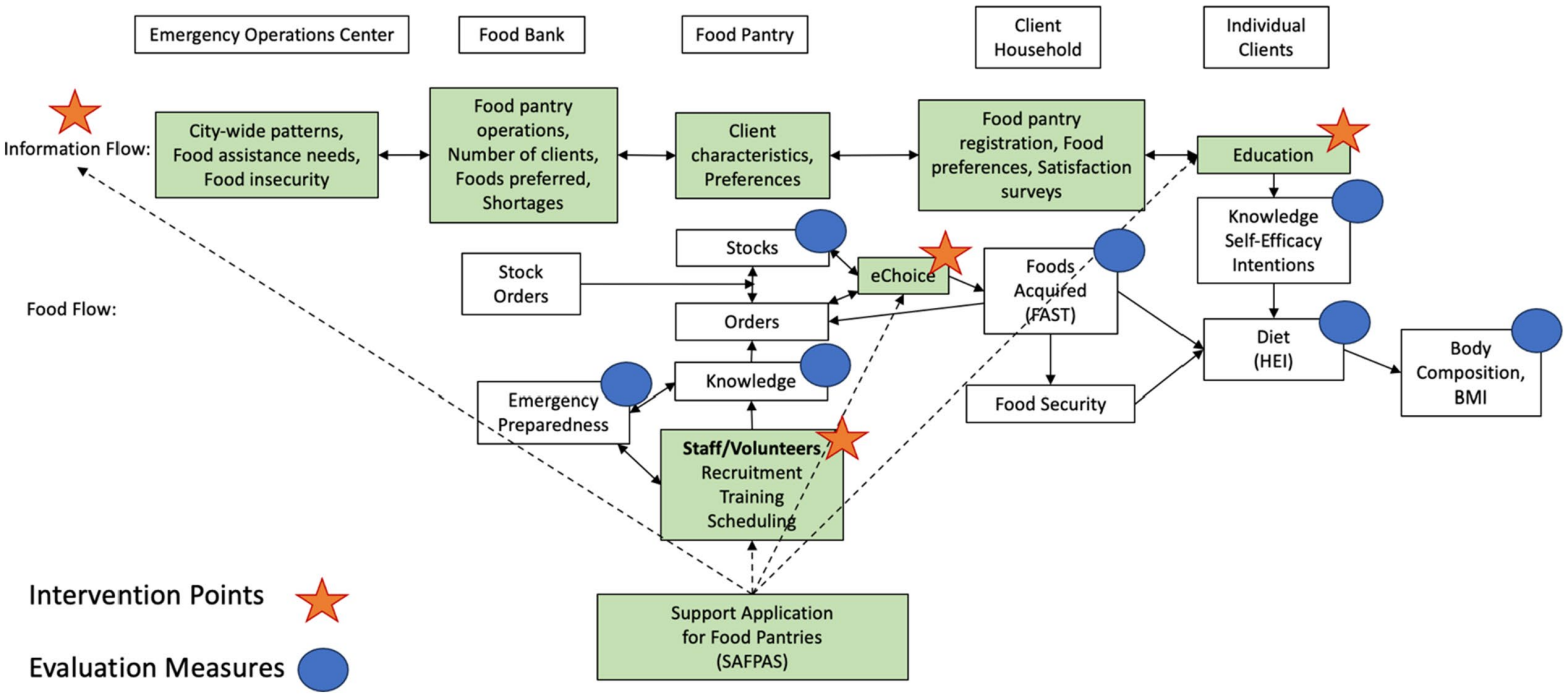
Conceptual framework guiding the SAFPAS trial. The green shaded boxes indicate SAFPAS app features. The intervention points are labeled with orange stars, and the evaluation measures (pre- and post-trial) are indicated with blue circles. HEI, healthy eating index; BMI, body mass index; FAST, food assessment scoring tool scores.

There are four main phases of data collection in the SAFPAS trial. First, we will conduct formative research in Baltimore, Maryland and Detroit, Michigan to refine the app prototype images and inform the development of the working app. Second, we will develop a stable working version of the app, and conduct functionality, stability, and usability testing (in Baltimore only). Third, we will employ a randomized controlled trial design (in Baltimore only) over a 12-month period to test the app and assess preliminary feasibility and impact at the food pantry and food pantry client levels. Finally, after the trial, we will conduct additional formative research in Baltimore and Detroit using the improved version of the app to prepare for scaling for use in different settings. The conceptual framework for the study is presented in Figure 1.

Evidence supporting the use of mobile apps to improve general health outcomes and disease management has been mixed; few studies have examined its utility among food insecure populations, and those which have are limited (15–17). However, authors note a lack of critical details about the development and user-centered design methods utilized to develop, and insufficient detail about testing the app in terms of usability, user-friendliness, and implementation (15, 16, 18). This study contributes to this growing area of the literature by providing app development and testing details, as well as by using a randomized-controlled trial to assess initial impact in a population experiencing food insecurity. Several other studies have employed a randomized-controlled trial to evaluate mobile apps (15, 16, 18–22).

### 2.2 Setting

Baltimore is the largest city in Maryland and is estimated to have nearly 570,000 residents, of which 61.6% are Black (23). Prior to the COVID-19 pandemic, 18% of residents experienced food insecurity, increasing to 21.7% following the COVID-19 pandemic (24). The Maryland Food Bank (MFB), founded in 1979 as the first food bank on the US east coast, is headquartered in Baltimore County, Maryland and supplies more than 300 food pantries, serves over 1,220 partners (soup kitchens, food pantries, shelters, etc.) across the state, and distributes more than 102,000 meals daily.

Detroit is the largest city in Michigan and has a population of 620,000 residents, of which 78% are Black (25). Estimates of food insecurity in Detroit vary, ranging from 30–70% of households (26, 27). Two food banks serve the Detroit area: Gleaners Community Food Bank and Forgotten Harvest (28). Gleaners works with more than 400 partner agencies and distributes over 47 million pounds of food annually (29). Forgotten Harvest has over 200 partner agencies and distributes 42 million pounds of food annually (30).

In order to optimize the app for use in low-resource contexts, we focused on both front-end and back-end improvements (31–37). For the front-end, we prioritized a lightweight, responsive design using React Native, HTML, and CSS, ensuring compatibility with older mobile devices and reducing resource consumption. On the back-end, we employed techniques such as API tuning, compression, caching, and server-side rendering to enhance performance and minimize data transfer. Cloud infrastructure from providers like AWS or GCP further supports scalability and availability. To address intermittent internet access, we implemented offline capabilities using PouchDB and CouchDB, enabling data storage and synchronization when connectivity is available. These efforts aim to overcome technology limitations and improve user experience (31–37), with findings informing future initiatives in similar environments.

### 2.3 Formative research

#### 2.3.1 Formative research prior to funding

This study builds upon extensive prior formative research, including sharing a series of low- and high-fidelity wireframe mockup images designed collectively by the research team with food pantry directors, previous intervention work in Baltimore food pantries (7, 38), two scoping reviews of the literature (12, 13), and a national survey of food pantry directors (14). Across this work, we found that food pantries in Baltimore experienced challenges related to finding a sufficient number of staff/volunteers, provide client choice and support clients in making healthy choices, and lacked emergency preparedness and response training. Among our food pantry partners and study participants, we found widespread support for a multi-featured app that would permit food pantry leaders to track pantry statistics, enhance all aspects of managing staff/volunteers and clients (called ‘neighbors’ in Maryland, and are referred to as such in the app) under both normal and emergency situations, and provide a remote and therefore safer version of client choice.

#### 2.3.2 Additional formative research

The first phase of this trial is additional formative research in Baltimore and Detroit to aid the design of the app. This includes interviewing food pantry staff and volunteers, and clients in both locations, and interviewing key staff from city agencies in Baltimore. We will purposively recruit food pantry directors, volunteers and staff from Baltimore (*n* = 12) and Detroit (*n* = 8); regular food pantry clients from Baltimore (*n* = 20) and Detroit (*n* = 15); and food bank personnel from the MFB (*n* = 6) and Gleaners Community Food Bank in Detroit (*n* = 4). In Baltimore we will also recruit key staff from city agencies such as the Health Department, Department of Planning, and Office of Emergency Management (*n* = 6). The sample size goals were generated based upon expected point of saturation given the specificity of the topic (39) as well as pragmatic considerations (e.g., budget and timeline constraints).

To be eligible, all interviewees must be at least 18 years old. Food pantry volunteers and staff must have worked with the food pantry for at least one year, plan to work at least one more year at the same food pantry, and have access to a smartphone or other device to access the web so they can use the app. Food pantry clients must be existing regular users of the food pantry as identified by the food pantry, must not anticipate moving out of Baltimore or Detroit for one year, have access to a smartphone, and be willing and able to use a web app through the smartphone to order food from food pantries. City agency staff must work for one of the relevant city agencies or food banks mentioned above. Interested and eligible interviewees will be invited to participate in a 45–60 min interview either via Zoom or in-person depending upon their preference. All interviewees will receive a $20 Visa Vanilla gift card for participating.

During the interviews, we will present high-fidelity wireframe mockup images of the different proposed features of the app, which provide snapshots of each app screen, to stimulate discussion around the look and design of the app, functionality, and what could be improved/changed about the app to better align with users’ needs. We will update the wireframe images iteratively based on these discussions. At multiple points throughout the formative phase, data collectors will present the feedback received from interviewees to the study team during weekly meetings. The research team will discuss each suggestion and decide whether to make the change/addition to the app based on data saturation and feasibility within the budget and timeline constraints of the study. If the team agrees the change applies to a wide range of pantries and is feasible, the wireframe images will be updated accordingly. The newly updated wireframes will then be used for subsequent interviews, and the process will begin again.

### 2.4 Randomized controlled trial

For the trial, which will only take place in Baltimore, we will recruit at the food pantry level. We will recruit 10 intervention and 10 control food pantries from a list of member food pantries provided by the Maryland Food Bank, foodpantries.org, listings maintained by colleagues who do work with food pantries, and The Maryland Food System Map list. Only medium and large food pantries will be recruited (>10,000 pounds of food distributed per year) given that small pantries may few staff, volunteers, and clients, and may be open infrequently. Food pantries that have participated in our trials previously will be excluded from participation, as will school- and health-center- based pantries given their potential different distribution systems compared to traditional food pantries. Once a pantry is recruited, we will recruit staff and volunteers (*n* = 100, 5 per pantry), and clients (*n* = 360, 18 per pantry) to be part of the trial using the same inclusion criteria described for formative research. These recruitment numbers are the goal reflect the results of a power and sample size calculation based on showing change in FAST scores.

### 2.5 Randomization of food pantries to intervention

Twenty Baltimore-based food pantries in this group-randomized trial will be first stratified by pantry size, established by the Maryland Food Bank (medium, >10,000 lbs. of food/year; or large, >25,000 lbs. of food/year), with half the pantries randomized to intervention, and half to comparison using a random number generator. Small pantries will be excluded given they may have few staff, volunteers, and clients, and may be open infrequently. By randomizing at the food pantry level, we aim to account for differences such as client population served, food distribution method, infrastructure (e.g., refrigeration), donations from other sources aside from the food bank, and staff/volunteer size.

### 2.6 App development process

We will develop a user-friendly and functional web-based app using the following processes (24–26). Figma software will be used to iterate the design of our user-interface by conducting user interviews to gain insights into the needs and pain points of food-insecure individuals and food pantries. This information will guide our development roadmap, ensuring the app addresses these challenges effectively. Our team of experienced developers leverages modern technologies and frameworks like JavaScript, and React Native to create an intuitive and visually appealing mobile user interface based on the final user-interface designs. We will prioritize scalability, security, and performance, integrating features such as user authentication, geolocation services, and payment gateways. Through iterative feedback loops with stakeholders, we can refine the app’s features and functionalities, resulting in a robust and user-centric solution that tackles food insecurity and enhances the user experience across the following domains:

*Define objectives and requirements*: The SAFPAS app development process begins with a collaborative effort to define clear objectives and requirements. Through extensive research and intended user engagement, we will identify key functionalities such as user registration, food donation, inventory management, scheduling, and notifications. These objectives and key functions of the app will be further refined throughout the formative research phase during interviews with key informants.

*Front-end development*: The term “front end” in the context of a web application pertains to the component of the software that users directly engage with during their interaction with the program. The term encompasses the various aspects of the user interface seen on a web browser or mobile device, such as its design, layout, and operation. In layman’s terms, the front end of a web application can be likened to the visual interface with which users engage.

*User registration and authentication*: We will implement user registration and authentication features to allow users to create accounts, log in, and securely access their profiles and personalized information.

*Functionality implementation*: We will develop the core features of the food pantry app based on the defined requirements, such as inventory management, scheduling food pickups, volunteer management, and notifications.

*Integration of third-party services*: We will integrate external services as needed, such as geolocation services for finding nearby food pantries or push notification services for real-time updates.

*Testing and quality assurance*: We will conduct thorough app testing to identify and fix bugs or issues. Perform functional testing, usability testing, compatibility testing across devices, and security testing to ensure a robust and reliable app.

*Deployment and app store submission*: We will prepare the app for deployment by generating appropriate build files for each target platform.

*Launch and user feedback*: Following deployment, the application will be made accessible to users and launched. In order to gain insight into the app’s functionality, performance, and potential areas for improvement, user input will be essential at this point. To improve the overall quality and user satisfaction of the app, it will be helpful to collect and analyze user feedback in order to find bugs, usability concerns, or other features that may be added in future updates.

We will incorporate user feedback forms within the app, allowing users to provide their insights, suggestions, and report any issues they encounter directly. Additionally, we will establish a user feedback loop through email communication and encourage users to reach out to us via email with any feedback, questions, or concerns they may have regarding the app. In our formative research after the trial, we will delve further into user feedback, preferences, and suggestions.

*Maintenance and updates*: App maintenance and updates are continuous processes that guarantee the app stays current, safe, and functional. In order to prevent vulnerabilities, this entails keeping an eye on the app’s performance, responding to any reported problems or bugs, installing security patches, and delivering updates that bring in new features or enhancements in response to user input. To maintain the app functioning properly and offer a satisfying user experience, regular maintenance will be necessary.

### 2.7 Functionality, stability, and usability testing

#### 2.7.1 Functionality and stability

Functionality and stability testing will be conducted using the following methods:

*Independent and integrated testing*: Each application component will undergo independent testing to ensure that defined inputs result in the expected outputs. Furthermore, integrated testing will be carried out to guarantee fluid communication and uniform software behavior throughout all components.

*Scenario-based testing*: To ensure consistency in functionality and compatibility, scenarios will be created to assess the system’s performance on various devices. Several use cases and user interactions will be covered in these scenarios to test the system’s performance in real-world circumstances. Functional test scripts and checklists will be created to validate all expected system components methodically. These scripts will cover a variety of capabilities and use scenarios to ensure the application satisfies the specified criteria and performs as expected.

*Stress testing*: Stress testing will evaluate the SAFPAS app’s stability and durability under adverse circumstances. Such tests include simulating user errors, such as accidentally clicking the “back” or “home” button. The app will undergo testing to ensure it is stable and will not crash under these conditions.

*User-error situations*: Various user-error situations will be outlined and tested against. Such errors differ from the ones considered in stress testing in that they originate from a lack of understanding of the app’s function rather than accidental input errors. Assessing user-error situations will make it easier to spot possible problems and guarantee that the app offers suitable feedback or gracefully manages mistakes without sacrificing stability or usability.

*Speed and responsiveness testing*: Using different types of connectivity, including Wi-Fi, cellular data, and hotspots, the SAFPAS app’s performance will be assessed. This testing will evaluate the app’s ability to provide a seamless user experience over a range of network environments.

The research and development process for the SAFPAS app will comply with industry standards by utilizing these improved testing techniques, assuring thorough testing coverage and resilience.

#### 2.7.2 Usability

The following strategy will be used to guarantee the app’s usability:

*Client-centered design evaluation*: The app’s user interface will be assessed using client-centered design techniques. User-centered design is the gold standard for app development and is critical to creating effective and engaging app experiences (40–43). The evaluation procedure will involve 27 participants (total combined participants from pre- and post-evaluation) from Detroit and 10 staff/volunteers from food pantries in Baltimore to understand the needs and preferences of the app users.

*Learning curve and proficiency testing*: To ascertain how soon users become skilled in using the app’s features, user proficiency testing will be conducted on days 0, 1, and 3 after training. This testing will reveal how simple it is to learn new things and how well the app supports users’ duties.

*Repeated user-satisfaction tests*: After the app is released to trial participants, repeated user-satisfaction tests will be conducted at various intervals. Feedback will be solicited during the testing process to determine whether the app meets end users’ expectations and satisfies their needs. This feedback will be assessed to identify and prioritize areas needing improvement.

*Data gathering and accessibility*: The app will be set up to gather and store data on a central server. An Structured Query Language database will be utilized to guarantee effective data administration and accessibility and to facilitate data analysis on standard statistical software, such as Stata 16 (StataCorp. 2019. *Stata Statistical Software: Release 16*. College Station, TX: StataCorp LLC). The program’s overall data will be updated frequently to update users and study coordinators on the app’s status.

### 2.8 Intervention trial description

The trial will be conducted in 3 stages (4 months each), differentiated by the types of SAFPAS features activated (Table 1). During the first weeks of each stage, formal training of participating food pantry directors/staff and MFB staff will take place – focusing on the use of any new features. Initial training will be followed up by proficiency testing in which pantry staff will be requested to complete certain functions (e.g., register a new client, locate an order, send a message). With each subsequent stage, additional app features will be made available to users to test. We will take this phased approach to introducing app features to allow time for training users on each feature; and by introducing a few features of the app at a time, we will have the opportunity to receive feedback and update the app as needed (see Table 1).

**Table 1:** SAFPAS app feature implementation phases by user type.

SAFPAS user	Phase 1	Phase 2	Phase 3
	Features introduced		
Client	Volunteer application, Pantry locator, Nutrition education messaging, Communications center	eChoice	Receive emergency information
Pantry staff and volunteers	Staff and volunteer management, Staff and volunteer training, Communications center, Information dashboard	eChoice	Bi-directional emergency information
Maryland food bank/City agency staff	View/access training materials, Communications center, Information dashboard	eChoice	Bi-directional emergency information

The SAFPAS app will have three main modules based on the three different intended user types: clients, food pantry staff and volunteers, and the Maryland Food Bank/city agencies.

#### 2.8.1 Client-facing features

Clients will be able to access the following app features:

*Volunteer application*: Clients can sign up to volunteer at their pantry through the app. Their “application” to volunteer will be reviewed by the pantry coordinator and can be accepted or rejected. If accepted, they will be able to view available timeslots and sign up for a shift.

*Pantry locator*: This feature will show clients where there are food pantries nearby. In addition to location, they can also view hours of operation, whether they are accepting new clients, whether volunteers are needed, and whether certain food items are in stock.

*Communications center*: Food pantries and city agencies will be able to push messages to clients in the communications center. These would include pertinent messages related to food pantry operations (e.g., closures due to weather) and emergency preparedness updates and information. This will allow clients to receive real-time updates on the status of their community food pantry.

**Figure 2:**
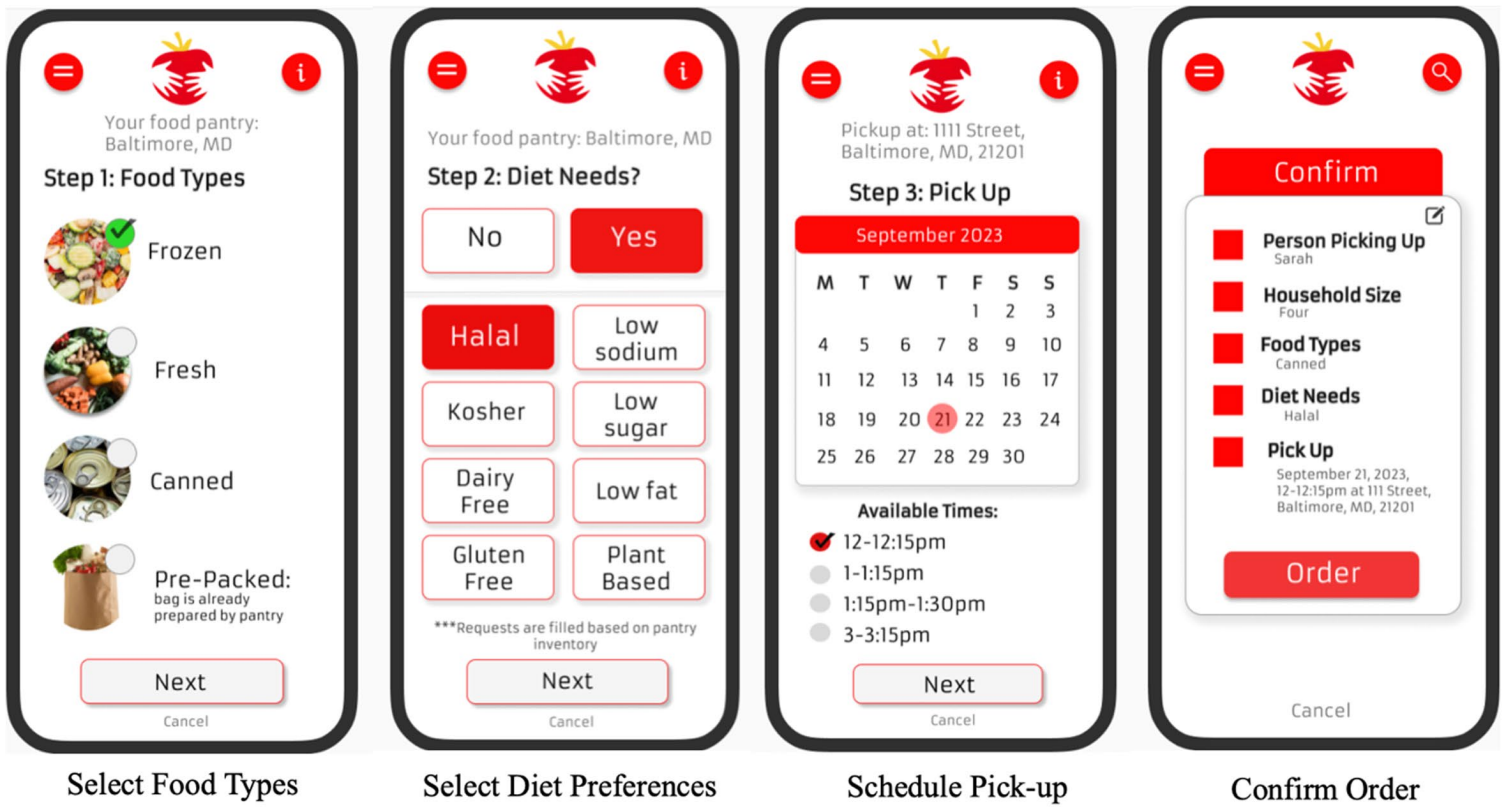
SAFPAS eChoice feature wireframe images.

*eChoice*: Clients will be able to use the app to create general food orders using the eChoice module (Figure 2). While clients will not be able to order specific foods, they will be able to order the types of desired food items (e.g., fresh, frozen, canned), select diet preferences in line with the USDA Dietary Guidelines for Americans (44) (e.g., heart healthy, vegetarian/vegan, Kosher, Halal, gluten free); and indicate food allergies. Pantries will be able to select which of these options are available to their clients. They will also have the option of selecting a pick-up date and time from choices identified by the pantry. While some pantries may not be able match client preferences, the data collected from these forms will be reported to the food bank and other stakeholders and will allow for better insight into what food types are most relevant in each community. This eChoice ordering system support client access to healthy choices, as it will allow clients to choose what foods fit their households the best based on their diet needs and preferences.

*Nutrition education*: Clients can opt to turn on nutrition education messaging to accompany their online ordering process. The feature will be informed by the Dietary Guidelines for Americans (44) and further developed during the formative research phase and client responses to baseline data collection questionnaires related to nutrition knowledge, self-efficacy, and intentions to meet client-specific needs. Messages will involve pop-up messaging and nudges related to specific dietary needs and chronic conditions such as cardiovascular disease (e.g., low sodium options) and diabetes (e.g., carbohydrate control). This feature is intended to enhance the healthfulness of foods chosen by clients, as they will repeatedly see helpful tips on which foods to opt for or avoid with certain health conditions.

*Emergency information*: Food pantry leadership and city agencies will have additional features to monitor real-time metrics from clients using the app. Dashboard views will be available and integrate into other Emergency Operations Center systems showing client requests, food pantry inventory levels and pantry-level requests for additional resources. City-level emergency management staff can also make use of the app’s messaging features to communicate to the pantries and distribute critical event information.

#### 2.8.2 Food pantry staff/volunteer-facing features

**Figure 3:**
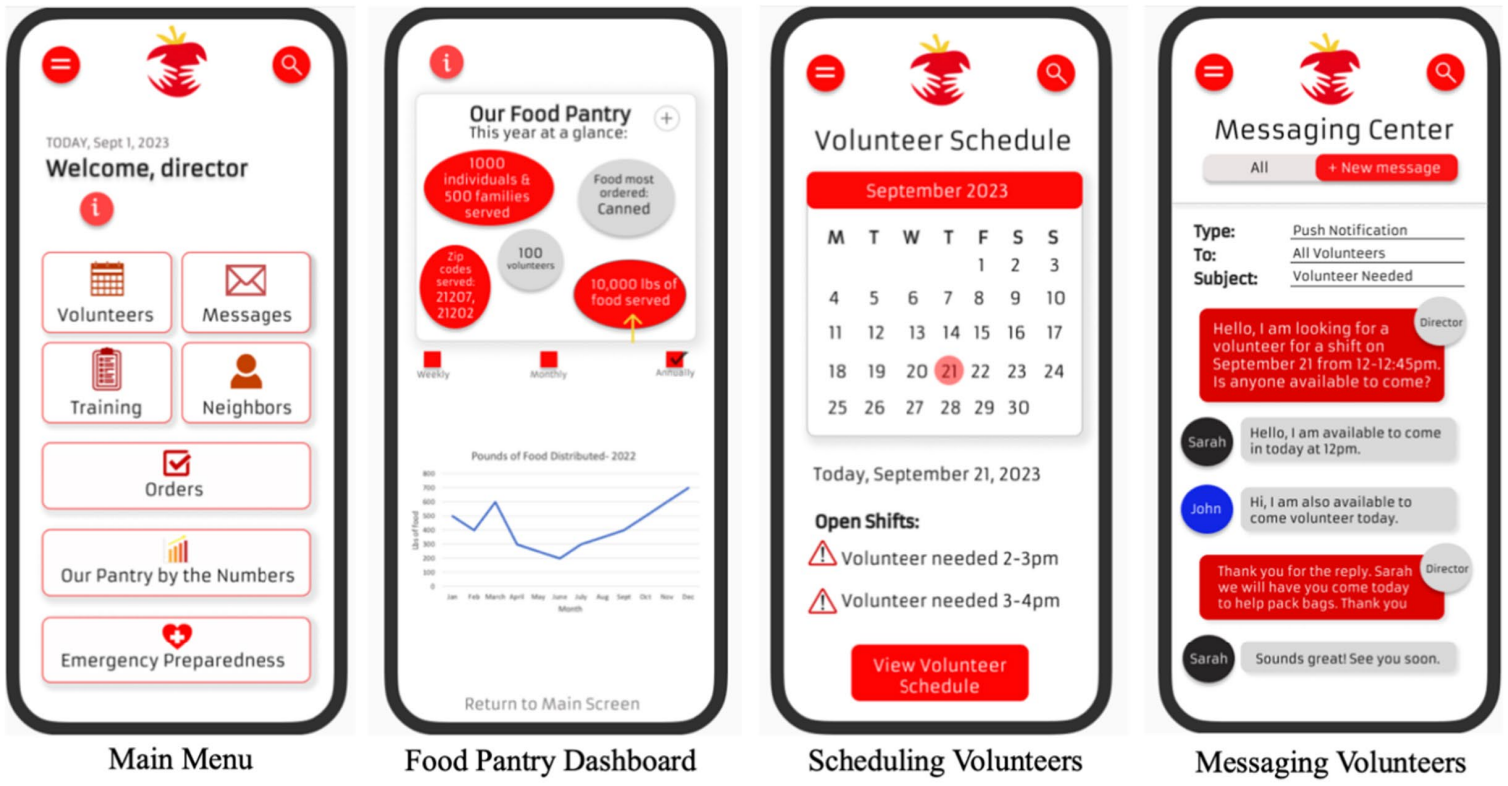
SAFPAS staff and volunteer management and communications center wireframe images.

Food pantry staff and volunteers will be able to access the following app features:

*Staff/Volunteer management and communications center*: Food pantry directors will be able to recruit, train and schedule volunteers through the app. The scheduling feature will include a calendar of shifts, open and filled slots, contact list of active and inactive volunteers, and status of volunteer training (who has completed, is in process, or has not started required training). This module will be integrated with the communications center, so directors can easily push notifications to volunteers or clients. Messaging may include schedule changes, emergency preparedness updates and information, and notifications about needs for volunteers (e.g., when a large delivery food arrives to the pantry) (Figure 3). This feature will streamline food pantry operations, allowing for volunteers and staff members to spend more time assisting clients’ access to healthy foods.

*Staff/Volunteer training*: In our previous formative work, we learned that food pantries currently have no streamlined way to train their new staff and volunteers, aside from mostly “on the job” training, which can be burdensome to the trainer (14). In response to this, there will be a series of training modules included in the SAFPAS app that can be completed by food pantry staff and volunteers, such as food safety and hygiene practices, and nutrition education.

*Information dashboard*: Pantry directors will be able to collect de-identified information from clients, volunteers and staff. Data will be automatically displayed and visualized via the Our Pantry Dashboard, which will include client statistics, real-time emergency situation information, and volunteer management (e.g., number of trainings completed) (Figure 3). This dashboard will also display user feedback on the eChoice system and other indicators of client satisfaction. These data can be exported to the food bank and other stakeholders, including local city agencies, in different report formats.

#### 2.8.3 Maryland Food Bank and city agency staff-facing features

**Figure 4:**
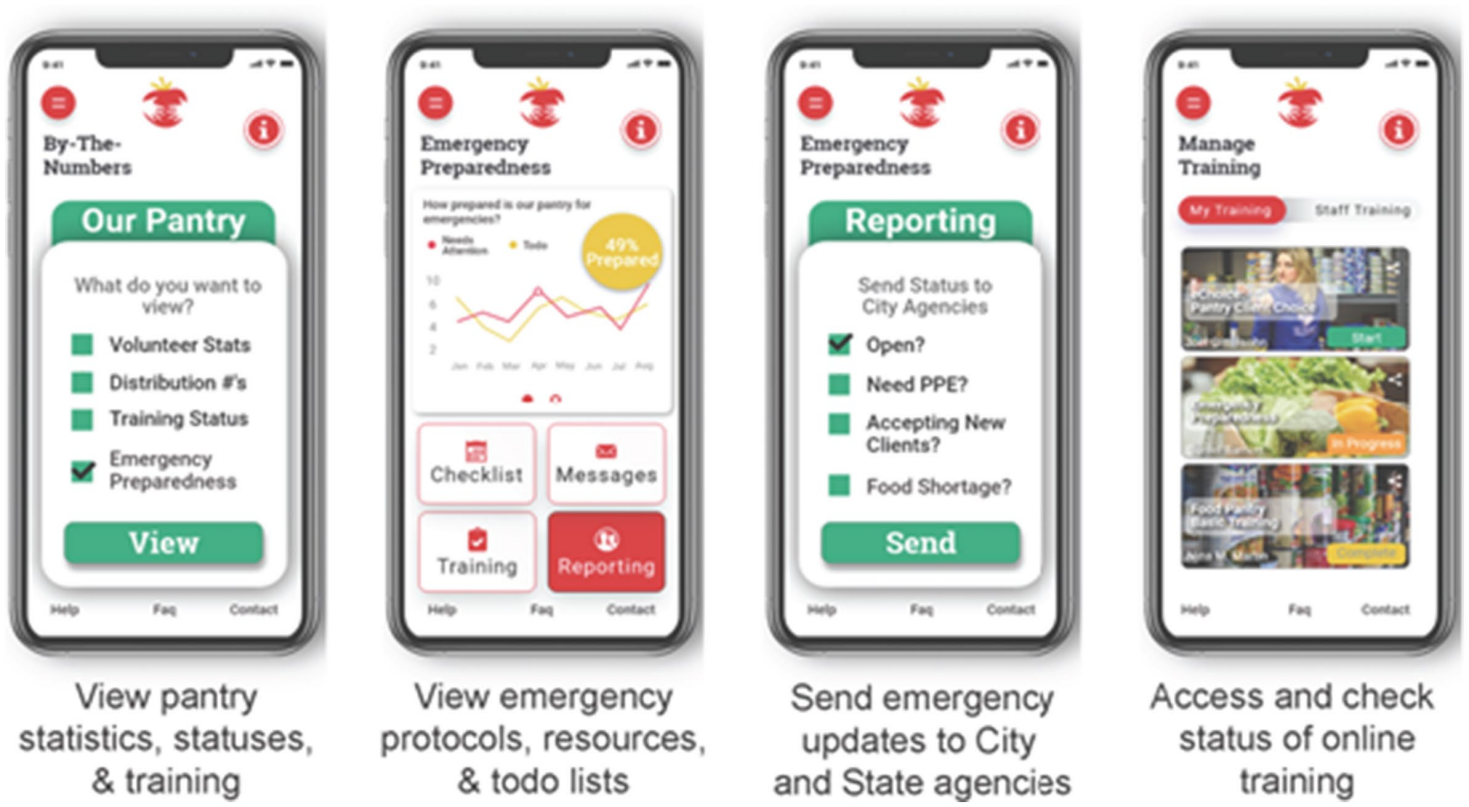
Dashboard, emergency preparedness, and training wireframe images.

Maryland Food Bank and city agency staff will have access to trainings, communications center, and dashboard features. The main menu for food bank users will be data oriented to provide quick access to statistics on food pantry clients, managers, and staff/volunteers who use the app (Figure 4). Staff can quickly send messages to food pantries (from a specific contact list or to all food pantry-related users). Last, there will be quick access to training materials and training status of all volunteers and staff, in order to permit revision and updating.

Data from the SAFPAS app will be available for sharing with governmental agencies for use in tracking and monitoring emergency situations. During an emergency activation, National Incident Management System best practices dictate units supporting the response routinely report their status and needs to the Emergency Operations Center (EOC). EOCs then conduct accountability for all external resources and facilities (i.e., food pantries), coordinate obtaining additional resources, and report upwards to the multi-agency coordination group, typically senior government leaders, on the overall status of the incident.

SAFPAS will provide a system where food pantries and banks can provide a daily “strength” report to the EOC. The granularity of such a report will depend on local needs. For example, a food pantry could report the number of clients, food insecurity status, food preferences etc., on a daily basis through a web interface. These data would be stored on a server managed by the EOC. At the EOC level, a dashboard will show supply at each participating food pantry. During an acute utilization time period, food pantries would likely need to report their status at increased intervals (e.g., hourly, every 3 h) to ensure information in the EOC is current. With the situational awareness information from this dashboard, the EOC could push updates further downstream to increase the efficiency of the food distribution center utilization. This dashboard would allow for rapid, timely review and increased overall efficiency.

### 2.9 Comparison group

The 10 food pantries in the comparison group will be introduced to the SAFPAS app after the intervention, including post-intervention data collection, is complete.

### 2.10 Trial evaluation

The SAFPAS trial will be evaluated at multiple levels, at baseline and post-SAFPAS intervention. The primary outcome is change in client Food Assessment Scoring Tool (FAST) scores of the food received from participating food pantries, and differences in changes in the score between the intervention and comparison groups. We will collect a series of secondary measures at the client, food pantry, food pantry staff and volunteer levels, app feasibility metrics, and process measures outlined. The measures will be collected by trained graduate research assistants and data collectors from the Johns Hopkins Bloomberg School of Public Health. Client-level participants will receive up to $40, food pantry managers/directors will receive up to $20, and food pantry staff/volunteers will also receive $20 up to two times each following data collection at baseline and post-intervention. They will receive this amount in the form of a Visa gift card.

#### 2.10.1 Client-level instruments

The FAST instrument consists of 13 categories of food and beverages. The total weight of each category is measured and used to calculate an overall score ranging from 0–100, which also takes into account the healthfulness of each food category. While the tool was originally designed to measure the overall healthiness of foods offered at pantries, it can be used to measure the healthfulness of individual client bags as well (45). In our previous Fresh Shelves, Healthy Pantries trial (FSHP), we found statistically significant improvements in FAST scores of pantry client bags in intervention pantries of more than 15 points, when compared with control pantries (38).

The Client Impact Questionnaire (CIQ) will be a modified version of a survey that we have used in previous studies with low-income Baltimore residents (46). This instrument will collect sociodemographic information (age, sex, income, food assistance program participation, food pantry usage, etc.), psychosocial measures derived from social cognitive theory (SCT) (e.g., knowledge, self-efficacy, and intentions) and food-related behaviors. The CIQ will include the Six-Item Short Form from the U.S. Household Food Security Survey (47) to capture food security at the household and adult level.

In addition, we will collect basic anthropometric measures. Body weight to the nearest 0.1 kg and body composition (% body fat and lean mass) will be measured with Bioimpedance Analysis (BIA) using the Tanita-BIA (InBody 270 body composition analyzer). Height to the nearest 1 cm will be measured with a stadiometer (Seca 213). Three measures will be made, and the closest two measures averaged. The CIQ will be performed at baseline and post-intervention (2 times) on the same sample of clients. All client measures will take place at the Johns Hopkins Bloomberg School of Public Health in a private office, or at their food pantry in a private location.

A validated Adult Block Food Frequency Questionnaire (FFQ) (2014) will be used to estimate client food intake and nutrient consumption (e.g., total energy intake, total fat, added sugar, sugar sweetened beverage and fruit and vegetable intake) (48, 49). Completed FFQs will be analyzed by NutritionQuest (Berkeley, California) and estimates of food patterns and dietary quality will be generated, including Healthy Eating Index scores (50).

#### 2.10.2 Food pantry staff and volunteer-level instruments

A Food Pantry Staff/Volunteer Impact Questionnaire will be developed to assess food pantry staff/volunteer knowledge and behaviors related to nutrition, food hygiene, and emergency management in about 100 participants (approximately 5 per pantry). Pantry managers/staff will be selected on the basis of seniority, with preference given to more senior staff, and those who intend to continue working in the pantry for at least one year. Questions will correspond to the information presented in the SAFPAS training modules and will be measured at baseline and post-intervention.

#### 2.10.3 Food pantry-level instruments

The Food Pantry Environmental Checklist (FPEC) was developed as part of the FSHP trial (7), and is used to assess the availability of healthy and unhealthy foods and beverages at food pantries. Foods and beverages are categorized based on the United States Department of Agriculture’s five groups: dairy, fruits, vegetables, grains, and proteins. Additionally, there are two more categories for high-sugar and high-fat foods and beverages. Along with food, the FPEC has a section on the food pantries’ policies on procuring, stocking and distributing food items, including questions regarding the number of freezers/refrigerators available and which specific programs donate food, among others (45).

We will use several existing questionnaires in our formative work to develop a pantry-level emergency preparedness questionnaire. We will utilize sections of the Centers for Disease Control and Prevention Crisis Emergency Risk Communications (CERC) Assessment Tool (51), EOC Position Checklists (52), and the Personal Protective Equipment Burn Rate Calculator (53), addressing behaviors in the emergency preparedness training module that will be developed, and based on input from the Baltimore Office of Emergency Management (OEM) to create a scale to assess emergency preparedness among pantries. The instrument will be piloted with a sample of approximately 10 food pantry directors.

#### 2.10.4 App feasibility metrics

The SAFPAS app will generate user statistics, including information on level/frequency of user engagement and who is using the app (e.g., clients, food pantry directors/staff, food bank staff). This information will be stored in a database and will be updated daily in a web-based dashboard. We will define user engagement variables and system-generated data to be captured and stored by the system for later analysis, including time(s) and duration of sessions, frequency of use and other technical data (battery charge, connectedness). In addition, select data will be accessible to users in a graphical form through the dashboard.

#### 2.10.5 Process evaluation measures

App-generated data will be applied to develop process evaluation measures – including the reach, dose delivered, and fidelity of the SAFPAS intervention implementation (54). The team will develop specific standards for each process evaluation component that will be generated through the SAFPAS app itself. For example, reach would include the number of clients and food pantries using the app; dose delivered would constitute the number of trainings completed by food pantry volunteers; and fidelity would reflect how fully the features of the app are being used. We will monitor our ability to meet set standards, and work to improve as the trial proceeds. Research team members will meet weekly during implementation to discuss progress, address challenges, and adjust delivery.

### 2.11 Sample size and power

A sample size calculation was conducted to assess the impact on Aim 3: the change in the healthfulness of client bags (primary outcome). The calculation was based a cluster-level t-test comparing the mean change in FAST scores of client bags at the intervention pantries to those at the comparison pantries. In our calculation, we used the reported mean change in FAST scores in comparison and intervention food pantries from the previous FSHP trial (7) with that hypothesis that there will be a trend toward increased FAST scores among intervention pantry clients compared to clients who are sampled from comparison pantries. Ultimately, sample-size assumed a difference in FAST Scores of 12 points, effect size of 0.8, intraclass correlation of 0.2, type I error of 5%, and power level set at 80%, to lead to a total sample size of 20 stores with 10 per arm and 18 clients per pantry (*n* = 360 at baseline). One pantry was added to each arm of the study to account for possible attrition, leading to the final sample size of 40 pantries. Similarly, we have added 3 clients per pantry to allow for potential drop out and still have necessary power based on our previous trial (7).

For direct comparison of the change in outcome over the intervention period, we will calculate an individual’s change in FAST score over time for each outcome by subtracting the pre-intervention value from the post-intervention value. These changes will then be compared between the intervention groups using cluster-level t-tests or nonparametric Wilcoxon-Mann–Whitney tests, depending on the normality of these individual changes.

The study is not powered to test for differences in secondary outcomes (e.g., nutrition knowledge, BMI, dietary intake, food pantry environment); initial findings will be used as preliminary data for a larger trial.

### 2.12 Data analysis

Qualitative data produced during the formative research, usability testing, and post- intervention feasibility assessment will be transcribed, cleaned, and coded by trained research assistants using the Atlas.ti 22 textual data analysis software program. A coding scheme will be developed for the textual data and applied by trained coders. In combination with memoing and structured queries, we will use the Atlas.ti 22 program to identify key themes associated with each of the focal areas. An emphasis will be placed on comments/themes related to the feasibility (acceptability, operability, potential sustainability, etc.) and future usability of the application by food pantry, food bank and emergency operations directors and staff.

The statistical plan will begin with exploratory analysis to examine broad trends and intervention effects from pretest score/intake to posttest score/intake. We will compare the change in FAST scores between intervention and comparison groups using cluster-level t-tests for normally distributed continuous changes or nonparametric Wilcoxon-Mann–Whitney tests for non-normal continuous changes. Then we will conduct linear mixed-effect models for confirmatory testing of intervention effects, with adjustments. T-tests will be used for normally distributed continuous variables and nonparametric Wilcoxon-Mann–Whitney tests for non-normal continuous variables. We will follow the exploratory analysis with multilevel mixed models. The mixed effects regression models will use change in pantry- and client-level scores over time as outcomes and will be modified to identify intervention effects as well as risk factors in regression analyses. Adjustments for client demographics (age, sex, education, etc.) and pantry-level variables (e.g., pantry size) will be considered. The clients’ “home” pantry will be treated as a random effect. It is possible that our client sample will use multiple food pantries, leading to possible contamination. Thus, we will calculate an overall intervention pantry exposure score and adjust our analyses. However, we anticipate contamination will be mitigated by giving app access to the 18 selected intervention pantry clients only. Posttest scores will be response variables in the models. The primary hypothesis is: compared with comparison pantries, client bags in intervention pantries will have a greater improvement (larger positive change) in FAST scores from pre-intervention to post-intervention. We will compare the mean change in FAST scores of participant bags from the intervention pantries to those from the comparison pantries. We expect that the SAFPAS intervention will result in a mean improvement (positive mean change) in FAST scores that is 12 FAST points better than the mean change in the comparison group.

## 3 Discussion

This paper outlines the protocol for the SAFPAS trial, an innovative, scalable digital strategy to enhance pantry management and meet client needs during routine and emergency operations. This protocol includes critical details about app development and evaluation previously highlighted as a glaring research gap in existing literature (15, 16, 18). The SAFPAS app aims to promote healthy foods at multiple levels of the emergency food system network that is flexible to respond to emergency management requisites in times of need. The app encompasses many aspects of running a food pantry, and allows communication with a network of stakeholders, making it novel compared to existing apps. SAFPAS also includes an emergency preparedness and response component which was a gap highlighted during the COVID-19 pandemic. Employing this approach may increase uptake and sustainability among food pantries and clients, and may increase access to healthful foods in these urban populations.

Existing interventions at food pantries have largely focused on nutrition education and environmental changes to encourage clients to make healthier selections (55–58). None have targeted multiple levels of the system (clients, pantries, food banks, city agencies). Previous literature shows that food pantries want to increase and emphasize healthy foods, but often lack the resources to do so, due to various capacity constraints in the emergency food system (5–7, 38). Research surrounding the emergency food system and its capacity to meet the needs of its clients highlights a lack of alignment between the pantries and the very communities they are intending to serve (59). Having the ability to choose foods was clients’ top priority when getting food from pantries, yet meeting this demand is difficult at the pantry level (5). The SAFPAS app aims to fill this gap by facilitating bidirectional communication from the client, to the pantry, and to the food bank to better align supply and demand for specific food items, including during emergencies and disasters, that align with community-identified priorities and needs.

In the context of helping to mitigate food insecurity challenges during emergencies and disasters, this app will include a number of novel elements. To this end, the SAFPAS app will include food bank inventory information, allow for client and staff to engage in real-time messaging within the app, and provide clients with nutrition information. Food pantry directors will have a dashboard to display staff/volunteer training compliance, real-time information on clients, hours of operations, and administer nutrition education campaigns. Using an all-hazards approach, during an emergency (e.g., pandemic, weather emergency) the SAFPAS app will support and directly integrate with local EOCs, city health departments and emergency management leadership. By directly connecting the food pantries with the EOC, governmental leaders will have a timely source of information that can be integrated and analyzed against other disaster response and recovery needs (e.g., transportation, public works).

Future emergencies and disasters such as newly emerging pandemics, and increasingly frequent and severe extreme-weather events in the face of climate change, will pose ongoing risks to the integrated food system. While other aspects of the social safety net are focused on emergencies (e.g., traditional first responders), food pantries and food banks were not initially created with disaster response and recovery in mind. From a preparedness perspective, food pantries must accordingly be resilient, with agility to accommodate acute nutritional demands during a disaster and interface with the centralized governmental response. By directly linking the food pantries and food banks to EOCs, the SAFPAS app will give senior leaders timely access to pantry metrics to better integrate that information into their common operating picture. Simultaneously, clients will have situational awareness about how their needs can be best met, through directions to a food resource that can meet their nutritional needs and preferences.

### 3.1 Challenges and strengths

One limitation of the present trial is that it is being implemented in one city only due to budget limitations. However, we will also be conducting formative research and feasibility testing in Detroit. This is an initial step toward being able to scale the app to other settings in a future, larger trial. Challenges with scaling to non-urban settings or larger cities with more cultural diversity will be considered in future iterations of the app. Of note, food pantry-based food is about half of clients’ diets, making it challenging to identify the app’s overall impacts on clients’ diets (60). However, to address this challenge, we will ask about food-getting behaviors (including frequency) and assess dietary intake in an effort to assess impact.

In this initial trial, the app will only be available in English which will limit the types of users it serves. Use of the app also requires a smart phone and the means to pay for data usage which is a barrier for some populations. Similarly, cell service (or Wi-Fi) is required which may be limited in some disaster settings. The SAFPAS app will be created from grant funding under an NIH R34. Should the app be successful in this study, long-term questions about ownership, ongoing maintenance and continuing implementation will need to be considered, and the final SAFPAS app will be distributed freely as open-source code.

## 4 Ethics and dissemination

This intervention trial was approved by the Johns Hopkins Bloomberg School of Public Health Institutional Review Board (IRB) (#IRB00024583) and the Oakland University IRB (#IRB-FY2023-314). Informed consent will be obtained for all participants for the formative research and for the impact evaluation of the randomized controlled trial. Results from this trial will be disseminated through peer-reviewed scientific publications, conference presentations, social media, and to participating food pantries and city agencies via presentations or briefs.

## 5 Conclusion

We anticipate that the SAFPAS app will improve alignment in the supply and demand for healthy foods among food pantry clients, food pantry stock, food banks, and city agencies which supply food. The overarching goal is to improve the healthfulness of the foods received by food pantry clients. Real-time, bidirectional communication between entities across the system allows for increased situational awareness at all levels during normal and emergency operations. By conducting formative research in Detroit, we hope to increase the scalability of the SAFPAS app to other settings in the future.

## Author contributions

DB: Conceptualization, Funding acquisition, Methodology, Supervision, Writing – original draft, Writing – review & editing. SS: Investigation, Methodology, Project administration, Writing – original draft, Writing – review & editing. MR: Conceptualization, Methodology, Supervision, Writing – review & editing. AL: Investigation, Writing – review & editing. LP: Conceptualization, Investigation, Methodology, Project administration, Writing – review & editing. AR: Writing – original draft, Writing – review & editing. AO: Data curation, Investigation, Software, Writing – review & editing. TI: Conceptualization, Data curation, Methodology, Software, Supervision, Writing – review & editing. RN: Conceptualization, Writing – review & editing. CR: Writing – review & editing. EL: Writing – review & editing. LJ: Conceptualization, Methodology, Writing – review & editing. LM: Investigation, Writing – review & editing. VV-B: Investigation, Project administration, Writing – review & editing. BG: Investigation, Writing – review & editing. NA: Investigation, Writing – review & editing. JG: Conceptualization, Funding acquisition, Methodology, Project administration, Supervision, Writing – original draft, Writing – review & editing.
